# Supplementary material for: Herbicide injury induces DNA methylome alterations in Arabidopsis
Source: PeerJ. 2017 Jul 20;5:e3560. doi: 10.7717/peerj.3560 (PMC5522609; doi:10.7717/peerj.3560)
Supplement: Supplemental Information 1 — Overview of sequencing results, breadth of coverage, and identification of methylated cytosines (mCs) following bisulfite sequencing of gDNA collected from newly formed cauline leaves at silique maturation of four A. thaliana individuals from each treatment where four-week-old rosettes were exposed to 0, 5, or 10% of a typical field rate of 0.9 kg acid equivalency ha−1 glyphosate. [file peerj-05-3560-s001.docx]

**Table S1.**

Overview of sequencing results, breadth of coverage, and identification of methylated cytosines (mCs) following bisulfite sequencing of gDNA collected from newly formed cauline leaves at silique maturation of four *A. thaliana* individuals from each treatment where four-week-old rosettes were exposed to 0, 5, or 10% of a typical field rate of 0.9 kg acid equivalency ha^-1^ glyphosate. Percent mCs were calculated for each context, for example the percent mCs in the CG context are based on total Cs in that context (Thus, percentages do not sum to 100 across different contexts).

|  | |  |  |  |  |  |  |  | | **Methylation levels** | | | | | |  |  |  |
| --- | --- | --- | --- | --- | --- | --- | --- | --- | --- | --- | --- | --- | --- | --- | --- | --- | --- | --- |
| **Glyph. (%)** | **Replic.** | | **Samp. ID** | **Total # Reads** | **# aligned** | **Seq depth(x)** | **Total Cs** | **CG context** | | | **CHG context** | | | **CHH context** | | | **Total mCs** | **Non-conversion (%)** |
|  |  |  |  |  |  |  |  | **No. mCs** | **%** | | **No. mCs** | **%** | | **No. mCs** | **%** | |  |  |
| **0** | Rep1 | | C1 | 74,574,448 | 43,590,398 | 64.6 | 780,336,560 | 51,265,027 | | 43 | 22,869,805 | | 18 | 31,060,980 | | 6 | 105,195,812 | 0.69 |
|  | Rep2 | | C2 | 82,488,478 | 51,477,846 | 76.3 | 922,815,146 | 57,193,521 | | 41 | 27,391,172 | | 19 | 36,183,190 | | 6 | 120,767,883 | 0.42 |
|  | Rep3 | | N1 | 70,553,248 | 42,447,028 | 62.9 | 759,711,024 | 49,177,698 | | 43 | 23,918,895 | | 20 | 35,016,618 | | 7 | 108,113,211 | 0.41 |
|  | Rep4 | | N3 | 71,804,442 | 42,067,010 | 62.3 | 756,777,874 | 51,326,027 | | 44 | 25,442,766 | | 21 | 36,507,965 | | 7 | 113,276,758 | 0.39 |
| **5** | Rep1 | | B2 | 75,229,394 | 45,523,338 | 67.4 | 813,425,114 | 50,773,125 | | 41 | 24,117,073 | | 19 | 33,028,377 | | 6 | 107,918,575 | 0.40 |
|  | Rep2 | | J1 | 77,334,988 | 47,736,008 | 70.7 | 854,388,263 | 54,299,895 | | 42 | 26,432,060 | | 20 | 34,495,360 | | 6 | 115,227,315 | 0.42 |
|  | Rep3 | | J2 | 80,678,884 | 50,820,036 | 75.3 | 911,054,703 | 58,197,390 | | 42 | 28,641,400 | | 20 | 40,310,820 | | 6 | 127,149,610 | 0.58 |
|  | Rep4 | | L2 | 68,124,880 | 40,832,892 | 60.5 | 732,450,226 | 45,949,480 | | 41 | 20,796,441 | | 18 | 28,371,293 | | 6 | 95,117,214 | 0.85 |
| **10** | Rep1 | | D1 | 54,099,010 | 32,798,596 | 48.6 | 590,470,280 | 41,227,780 | | 45 | 22,193,872 | | 24 | 31,792,669 | | 8 | 95,214,321 | 0.47 |
|  | Rep2 | | D2 | 74,156,162 | 45,054,670 | 66.7 | 807,306,425 | 52,378,823 | | 43 | 27,374,902 | | 22 | 36,836,114 | | 7 | 116,589,839 | 0.45 |
|  | Rep3 | | P2 | 81,114,422 | 49,284,324 | 73.0 | 887,675,068 | 60,577,608 | | 45 | 32,400,638 | | 23 | 49,919,766 | | 8 | 142,898,012 | 0.37 |
|  | Rep4 | | P3 | 62,450,556 | 38,410,522 | 56.9 | 686,900,287 | 45,872,154 | | 44 | 26,531,007 | | 24 | 40,369,192 | | 9 | 112,772,353 | 0.39 |
