# Supplementary figures and images for: Herbicide injury induces DNA methylome alterations in Arabidopsis
Source: PeerJ. 2017 Jul 20;5:e3560. doi: 10.7717/peerj.3560 (PMC5522609; doi:10.7717/peerj.3560)

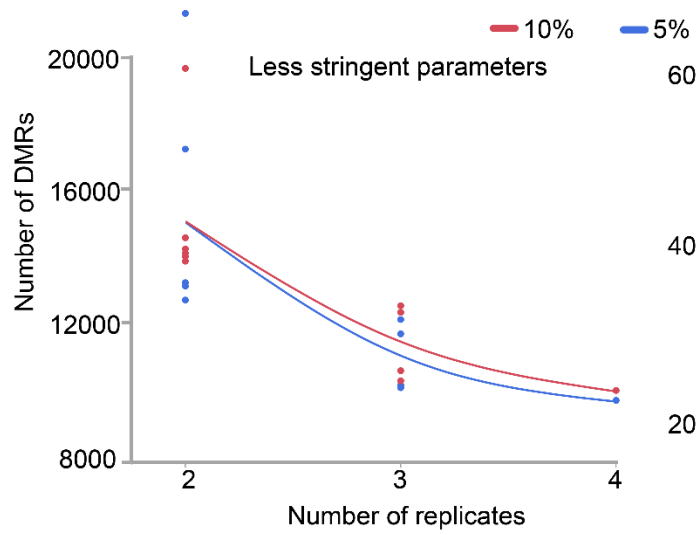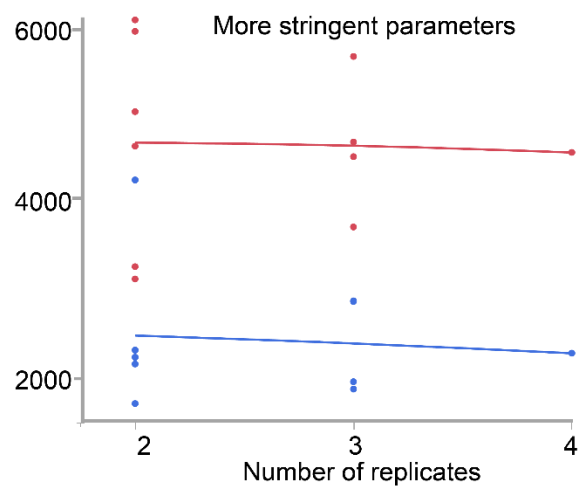

Supplement: Supplemental Information 2 — Effect of increasing the number of replicates in limiting the number of DMRs called by the BSmooth/bsseq R package (Bioconductor) using less stringent qcutoff of 0.1 (left panel) and more stringent qcutoff of 0.01 (right panel) parameters. The more stringent parameters and all four replicates were used to identify the DMRs in Dataset S3. Colored lines indicate 5 and 10% of a 0.9 kg acid ha−1 glyphosate rate. [file peerj-05-3560-s002.pdf]

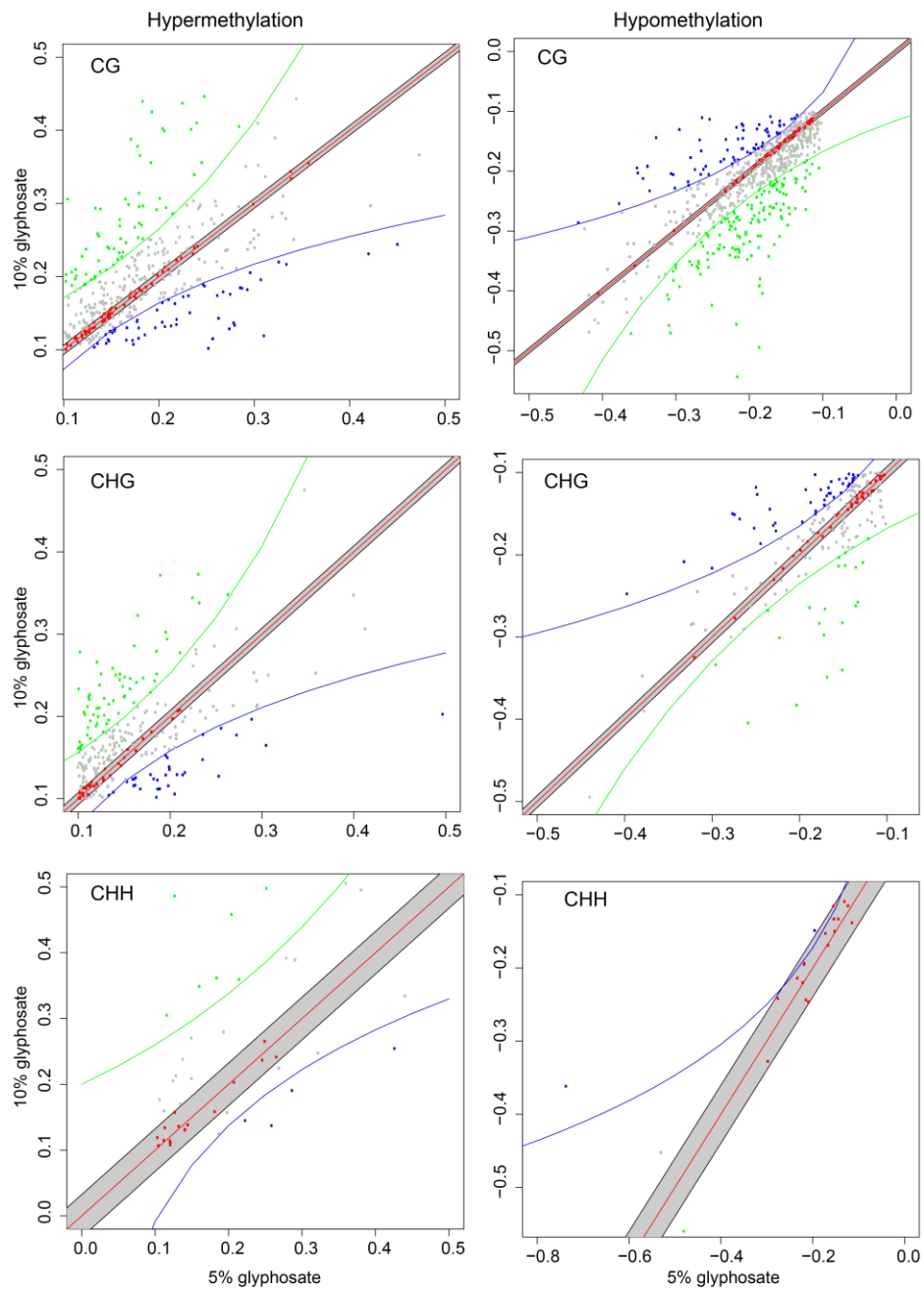

Supplement: Supplemental Information 3 — Categorization of overlapping DMRs identified following treatment at 5 and 10% of a 0.9 kg acid ha−1 glyphosate rate based on dose-dependency of methylation response. Using a 95% confidence interval cutoff, overlapping DMRs were classified as either doseindependent (red points), positive dose-dependent (i.e. larger methylation differences in the 10% glyphosate-treated plants than the 5% glyphosate-treated plants–green points), inverse dose-dependent (i.e. larger methylation differences in the 5% glyphosate-treated plants than the 10% glyphosate treated plants–blue points) or insufficient support for categorization (grey points). See Dataset S4 for list of DMRs categorized by this analysis. [file peerj-05-3560-s003.pdf]

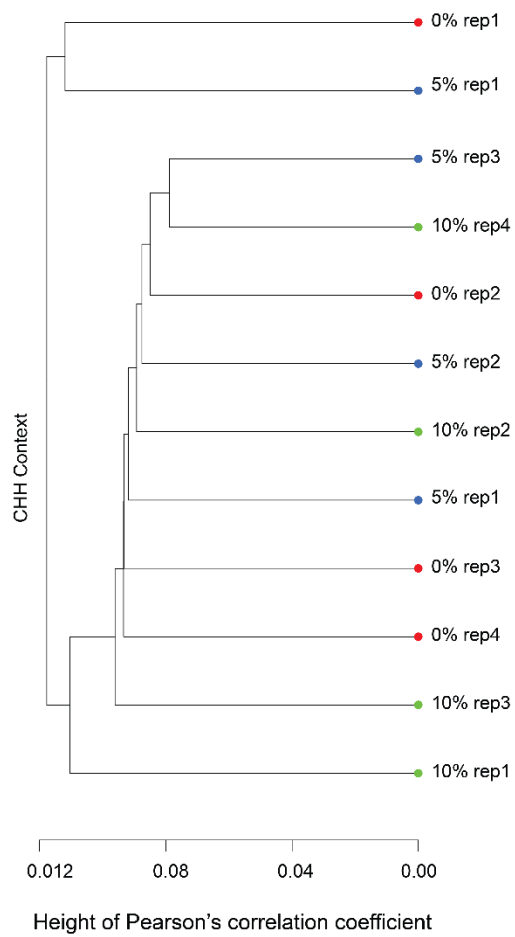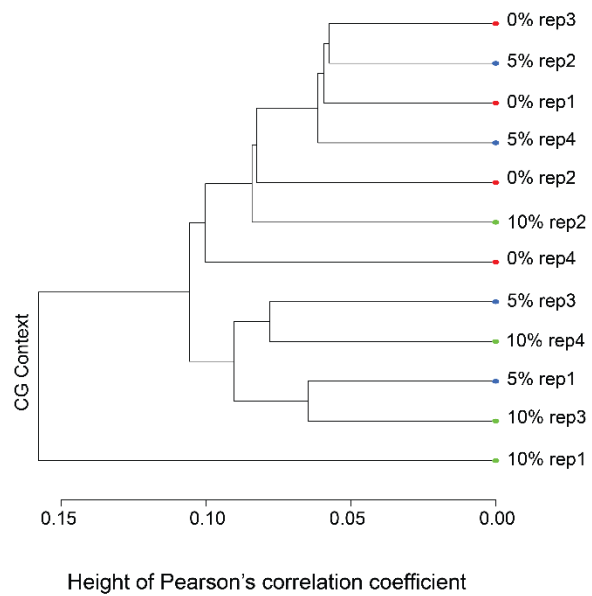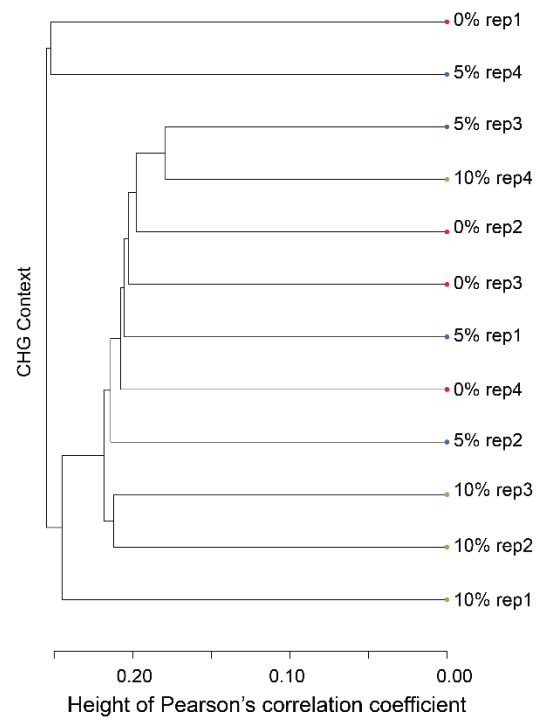

Supplement: Supplemental Information 4 — Treatments comprised percentages of a 0.9 kg acid equivalency ha−1 glyphosate rate applied to four-week-old A. thaliana rosettes. [file peerj-05-3560-s004.pdf]

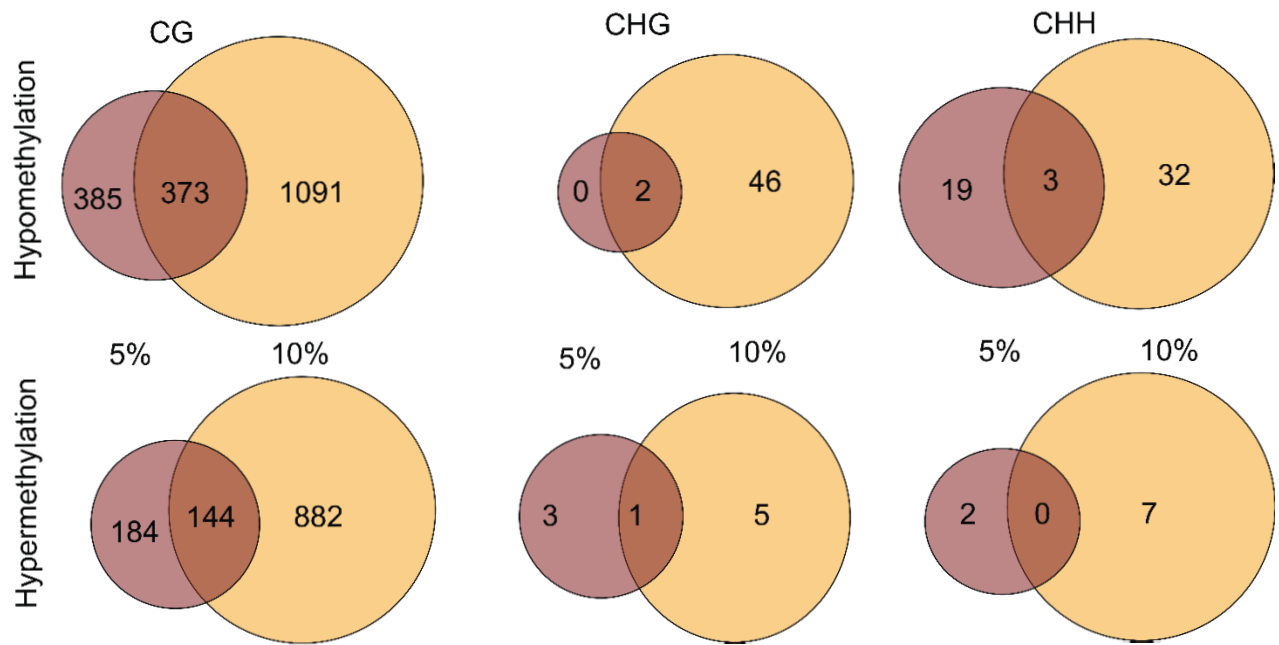

Supplement: Supplemental Information 5 — Number of DMRs identified using eDMR across the three sequence contexts and separated by hypomethylation vs hypermethylation and whether or not the DMR was identified in the 5% glyphosate-treated samples only, the 10% glyphosate-treated samples only, or in both treatment groups. See Dataset S2 for detailed list of all DMRs identified using eDMR. Glyphosate percentages based on an application rate of 0.9 kg acid equivalency ha−1. [file peerj-05-3560-s005.pdf]

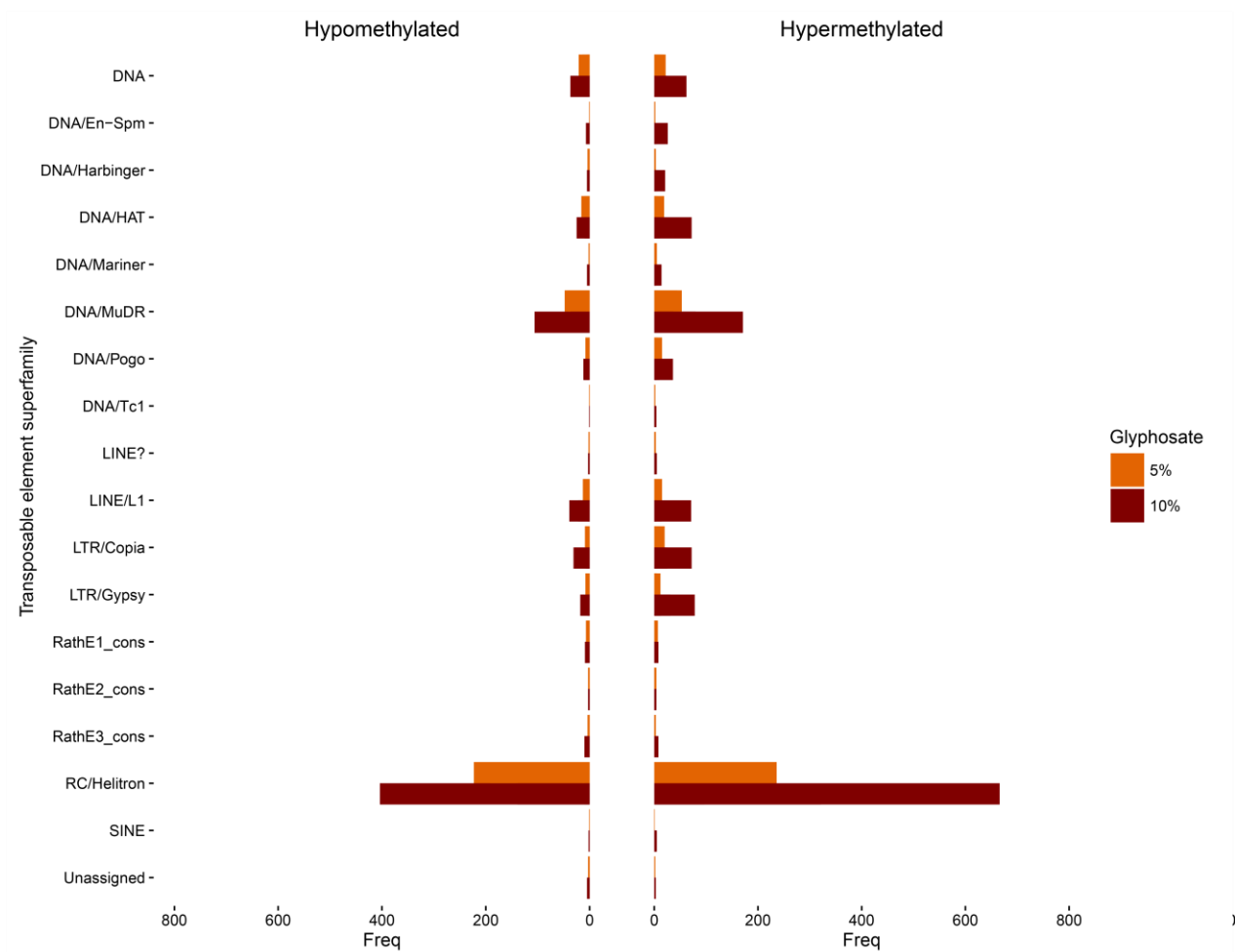

Supplement: Supplemental Information 6 — Frequency of DMRs associated with transposable elements superfamilies, identified from the TAIR10 genome release, across all contexts (CG, CHG, CHH). Panels separate hypomethylation vs hypermethylation and colors differentiate the 5% (orange) and 10% (maroon) glyphosate-treated samples. Methylation events are diverse across superfamily, treatment, and context. See Dataset S5 for a detailed list of all transposable element families and superfamilies associated with the identified DMRs. Glyphosate percentages based on an application rate of 0.9 kg acid equivalency ha−1. [file peerj-05-3560-s006.pdf]

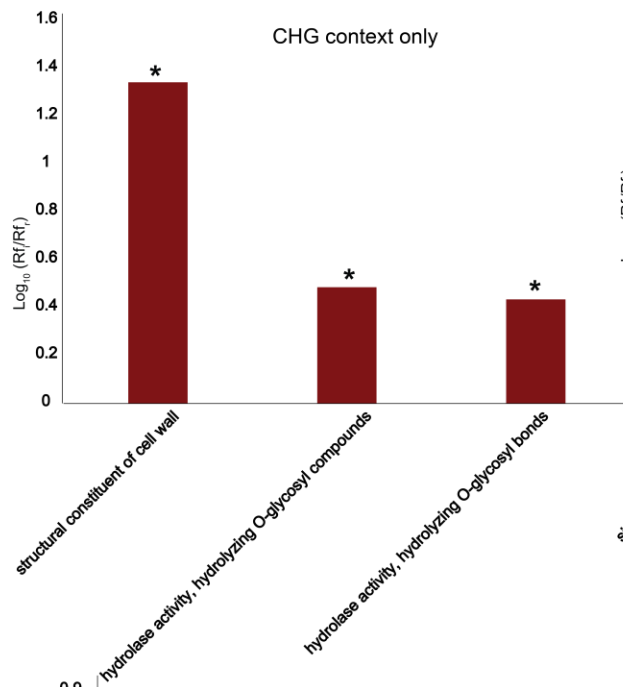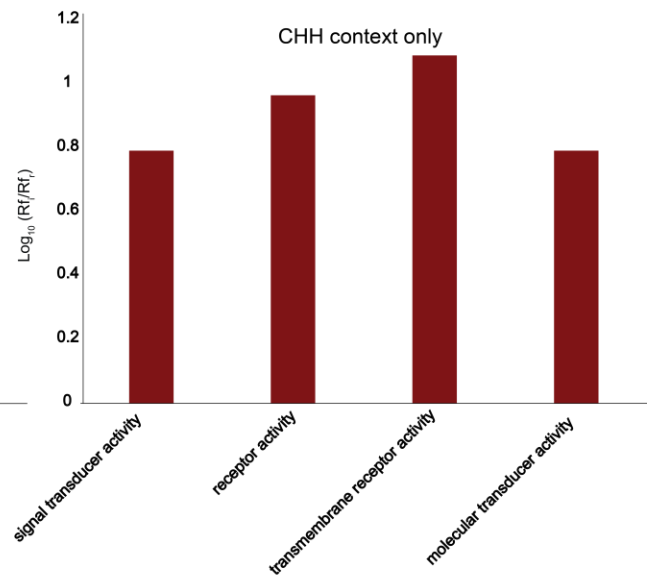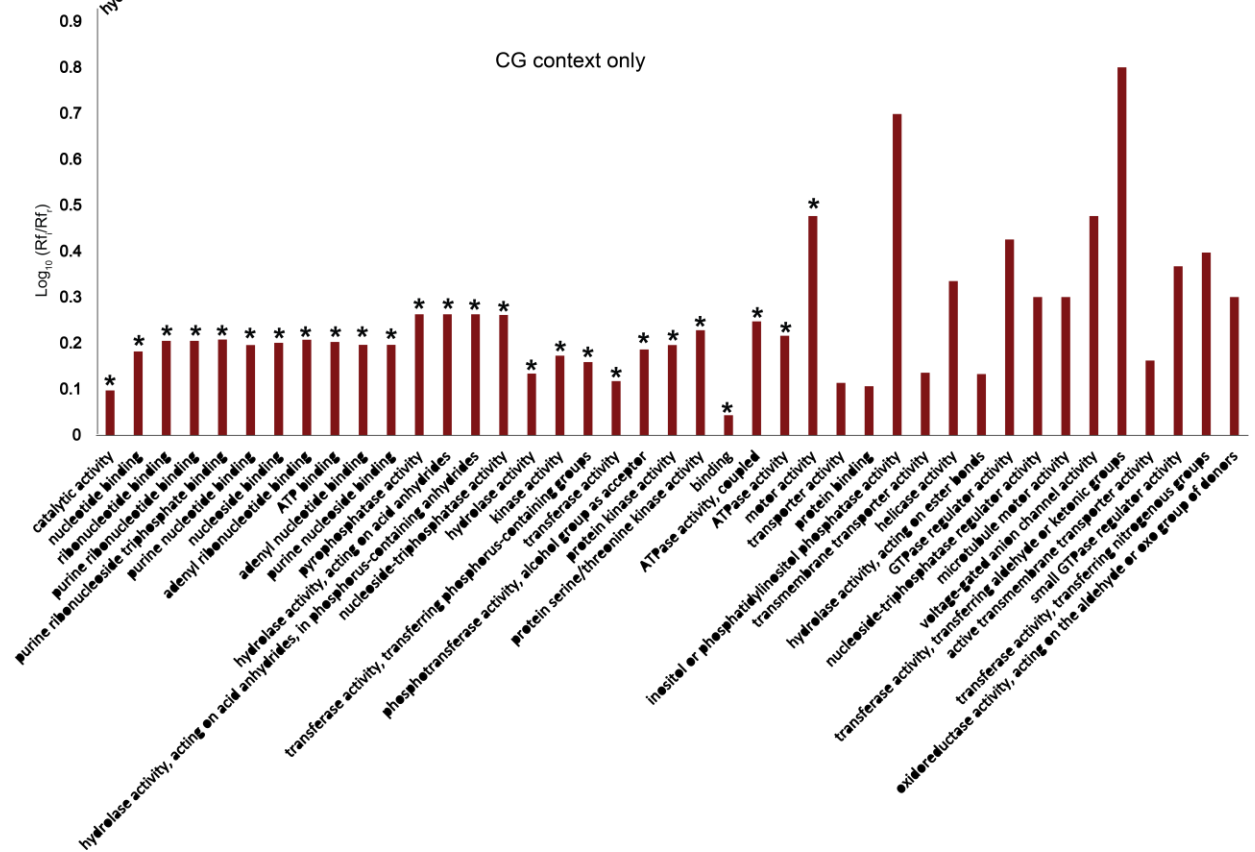

Supplement: Supplemental Information 7 — Gene ontology (GO) terms for molecular function enriched (p<0.05) in DMR associated genes for each of the three sequence contexts. Rfi/Rfr represents the ratio of the relative frequency of GO terms in the input (glyphosate DMRs) to the reference (TAIR10 Arabidopsis genome) datasets. * indicates p<0.01. See Dataset S6 for full list of GO terms with Rfi/Rfr >1.5 in each of the three sequence contexts. [file peerj-05-3560-s007.pdf]
