# Supplementary material for: Herbicide injury induces DNA methylome alterations in Arabidopsis
Source: PeerJ. 2017 Jul 20;5:e3560. doi: 10.7717/peerj.3560 (PMC5522609; doi:10.7717/peerj.3560)
Supplement: Supplemental Information 15 — Annotation of genomic features with DMRs. This custom Perl script identifies differentially methylated regions (DMRs) and provides genomic features annotation with TAIR10 gff file. [file peerj-05-3560-s015.rtf]

Supplemental Information.

Custom Perl script. Annotation of genomic features with DMRs. 

This custom perl script identifies differentially methylated regions (DMRs) and provides genomic features annotation with TAIR10 gff file. 
_________________________


#!/usr/bin/perl -w

# annotate the dmr with gene info.

use strict;

my $genefile = 'genelist';
unless (@ARGV)
{
	print "usage: perl $0 dmrfilename kbdistance\n";
	exit;
}

my $dmr = $ARGV[0];

my $kb = $ARGV[1];

open(IN, $ARGV[0]) or die "cannot open $!\n";

# dmr file is a bit messy! 
(my $outfile = $0) =~ s/pl/out/;
open(OUT, ">$outfile") or die;
# my $counter;
while (my $line = <IN>)
{
	chomp $line;
	if ($line=~ /chr\d/i)
	{
		# check the gene annotation
		my $geneinfo = annotate($line);
		print OUT $line,"\t", $geneinfo,"\n";
	}
	else
	{
		print OUT $line,"\n";
	}
	# $counter++; 
	# if ($counter>5) {exit;}
}
close OUT;

sub annotate {

	my $line = $_[0];
	my @dmr = (split/\t/,$line)[0..2];
	# check overlaped genes
	open(GENE, $genefile) or die;
	my $genes=''; # the string with all overlapped genes
	my %gene_reg; # the hash with gene and the specific regions
	my $overlap;  # the string to be returned with all the genes overlapping with the dmr region
	while (my $g = <GENE>)
	{
		# check each line for overlaps
		if ($g =~ /^$dmr[0]\b/i)
		{
			my @temp = split/\t/,$g;
			next if ($temp[2] =~ /chromosome/);
			my $regiontype = $temp[2];
			$temp[8]=~/AT\dG\d+/;  # id info.
			my $gid = $&;
			if (($dmr[1]-$kb>=$temp[3] and $dmr[1]-$kb<=$temp[4]) || ($dmr[1]-$kb<=$temp[3] and $dmr[2]+$kb>=$temp[4])
                || ($dmr[2]+$kb>=$temp[3] and $dmr[2]+$kb<=$temp[4]))
			{
				# overlap # gene info.
				if ($genes =~ /$gid/)
				{
					if ($gene_reg{$gid}!~/$regiontype/)
					{
						$gene_reg{$gid} = $gene_reg{$gid}.','.$regiontype;
					}
				}
				else
				{
					($genes)?($genes.=';'.$gid):($genes=$gid);
					$gene_reg{$gid} = $regiontype;
				}
			}
		}
    }
	# now print the overlap
	if ($genes)
	{
		my @genes = split/;/,$genes;
		foreach (@genes)
		{
			$overlap .= $_.':'.$gene_reg{$_}.";";
		}
	}
	else
	{
		$overlap = 'NONE';
	}
	return $overlap;
}
